# Is There Any Association Between Fat Body Mass and Bone Mineral Density in Patients with Crohn’s Disease and Ulcerative Colitis?

**DOI:** 10.3390/nu17030466

**Published:** 2025-01-28

**Authors:** Alicja Ewa Ratajczak-Pawłowska, Michał Michalak, Aleksandra Szymczak-Tomczak, Anna Maria Rychter, Agnieszka Zawada, Kinga Skoracka, Agnieszka Dobrowolska, Iwona Krela-Kaźmierczak

**Affiliations:** 1 Laboratory of Nutrigenetics, Department of Gastroenterology, Dietetics and Internal Diseases, Poznan University of Medical Sciences, 61-701 Poznan, Poland; arychter@ump.edu.pl; 2 Department of Gastroenterology, Dietetics and Internal Diseases, Poznan University of Medical Sciences, 61-701 Poznan, Poland; aleksandra.szymczak@o2.pl (A.S.-T.); aga.zawada@gmail.com (A.Z.); kingskoracka@gmail.com (K.S.); agdob@ump.edu.pl (A.D.); 3 Department of Computer Science and Statistics, Poznan University of Medical Sciences, 61-701 Poznan, Poland; michal@ump.edu.pl; 4 Doctoral School, Poznan University of Medical Sciences, 61-701 Poznan, Poland

**Keywords:** inflammatory bowel disease, osteoporosis, nutritional status, body fat mass, bone mineral density

## Abstract

**Background**: The study aimed to investigate the association between fat body mass and bone mineral density (BMD) of the lumbar spine (L1–L4), femoral neck, and total body. **Methods**: We studied 95 patients with Crohn’s disease (CD), 68 with ulcerative colitis (UC), and 40 healthy adults (control group—CG) aged 18–50 years old. The BMD of lumbar spine and femoral neck was assessed as well as body composition. **Results**: A lower fat mass percentage was observed in about 8% of CD, 13% of UC, and 3% of CG. An increased percentage of fat mass was common, and occurred above 50% of CD, 40% of UC, and about 60% of CG. Body fat mass and fat mass percentage were significantly lower among UC compared with the CG (*p*-value < 0.001) and CD (*p*-value < 0.01) in women. Body fat mass correlated positively with the BMD and T-score of L1–L4 and total body mass in men with UC. We found a positive correlation between the fat body mass and BMD and T-score of L1–L4, femoral neck, and total body in women with IBD. Among CG, positive correlations occurred between the fat body mass and BMD of L1–L4, BMD of total body, and T-score of total body, but only in men. CRP (C-reactive protein) correlated negatively with fat body mass only in men with CD. **Conclusions**: A higher fat mass percentage is common among IBD patients and healthy adults despite a normal body mass index. Body fat mass is a predictor of nutritional status and likely influences the course of the disease, as it correlated positively with BMD, T-score, and Z-score. The association between fat tissue and bone health appears to be stronger in women. Further studies are needed to investigate additional factors that may affect bone health in IBD.

## 1. Introduction

Malnutrition is a common complication of inflammatory bowel disease (IBD), affecting about 20–70% of patients. It is more prevalent in Crohn’s disease (CD) than ulcerative colitis (UC) [1]. Malnutrition leads to reduced body mass, resulting in both fat and fat-free mass loss [2]. Many factors contribute to malnutrition, including decreased intake, increased expenditure, surgery, and malabsorption [3]. Additionally, fat mass depends on the course of the disease, with its loss being associated with the active phase of CD or UC [4]. Moreover, Yadav et al. presented that fat mass decreases with increasing disease severity [5]. In patients with inactive CD, fat mass remains unchanged [6]. However, regardless of disease localisation, CD patients tend to have a lower fat mass than healthy subjects [7].

Fat mass is also influenced by treatment. The anti-TNF-α treatment led to an increase in fat mass among IBD patients [8].

Inflammatory bowel disease and osteoporosis are a group of diseases with multifactorial etiology. Of particular importance in their pathogenesis are immune, environmental, and genetic factors. Among the immune factors, it is vital to notice the role of cytokines, in which the main sources are monocytes and macrophages. They initiate and maintain the inflammation infiltrate in IBD. The main cytokine in IBD patients is interleukin-1α (IL-1α); interleukin-1β (IL-1β); interleukin-2 (IL-2); interleukin-6 (IL-6); interleukin-8 (IL-8); interleukin-12 (IL-12); interleukin-17 (IL-17); interleukin-23 (IL-23); tumour necrosis factor (TNF); and interferon (IFN). Additionally, interleukin-4 (IL-4); interleukin-10 (IL-10); and interleukin-13 (IL-13) are important in IBD. TNF, IFN, IL-2, IL-6, IL-8, are dominant cytokines in CD, and in UC—IL-4, IL-5, IL-10 i TNF. The inflammation process in IBD may also affect bone mineral density. The proinflammatory cytokine increases osteoclast activity and enhances osteoblast apoptosis [9]. This process is mediated by the OPG/RANKL/RANK pathway. The elements of this system are Receptor Activator of Nuclear Factor NF-κB (RANK), their ligand—Receptor Activator of Nuclear Factor NF-κB Ligand (RANKL) and osteoprotegerin (OPG), which decrease the RANKL–RANK interaction. This pathway is also regulated by cytokines and hormones: IL-1, IL-6, and IL-11 and TNF increase the OPG and RANK levels, and IL-6 decreases the concentration of RANKL. Therefore, IBD patients have an abnormal pathway activity, which promotes bone reabsorption [10].

Fat tissue is a source of cytokines, specific adipokines, and hormones. The main interleukins are IL-6 and TNF, which are also the main interleukins that affect bone metabolism. Additionally, in fat tissue, androgens are converted to oestrogens by aromatase. Estrogens play an essential role in bone turnover regulation. Estrogen inhibits osteoclast differentiation, decreasing the number of active rebuilt units. This action is regulated by IL-1 and IL-6 [10].

Malnutrition significantly impacts the quality of life and disease course. Moreover, malnutrition increases the risk of hospitalisation [11]. In hospitalised patients, malnutrition is also a risk factor for readmission and mortality [12]. It is important to note that undernourishment and insufficient intake increase the risk of low bone mineral density (BMD) [13].

According to the meta-analysis, the risk of osteoporotic fractures increases by 32% among patients with IBD [14]. The loss of bone mineral density is multifactorial and not fully understood. Additionally, the risk of osteoporosis development varies among patients with IBD [15]. The main risk factors of low bone mineral density in IBD patients are, among others, low body mass and BMI (body mass index), chronic inflammation, prolonged steroid therapy and malabsorption, especially vitamin D, calcium and vitamin K [13]. According to Harbord et al., to prevent bone loss, patients with IBD should quit smoking, maintain adequate calcium intake (1000 mg/day), and start wear-bearing exercise [16]. The main consequences of bone loss and low BMD are bone fragility and fractures. However, bone strength may also be affected by bone size, microdamage, or the microarchitecture of trabecular [17].

Identifying new factors associated with low bone mass will improve the diagnosis and treatment of osteoporosis in IBD and enhance the quality of life. Additionally, understanding new factors of osteoporosis may impact osteoporosis screening indications.

Our study focuses on fat mass, and does not include data on muscle mass. It aims to assess the association between fat mass and bone mineral density in patients with IBD.

## 2. Materials and Methods

In this prospective study, we included 95 patients with CD (women, n = 50), 68 with UC (women, n = 28), and 40 healthy adults (control group—CG), including 20 women, all aged between 18 and 50 years, recruited at the Department of Gastroenterology, Dietetics and Internal Medicine, Poznan University of Medical Sciences between 2020 and 2021. At the beginning of the study, we recruited 206 patients with IBD. However, some patients either did not consent to undergo DXA (Dual-energy X-ray absorptiometry) examination or had a co-existing disease that met the exclusion criteria. Inflammatory bowel disease patients were recruited during hospitalisation or standard doctor’s appointments. Patients included in the study had been diagnosed with IBD based on endoscopic, histopathological, and radiological criteria. All patients were treated according to the European Crohn’s and Colitis Organisation guidelines and the Polish Society of Gastroenterology. All subjects included in the study provided their written informed consent. The local Bioethics Committee approved the study (Resolution no. 39/20 of 16 January 2020). The exclusion criteria were pregnancy and co-occurring diseases which may affect BMD, including celiac disease, neoplasm, liver diseases, kidney diseases, diabetes, and rheumatoid arthritis. All subjects carried out Dual-energy X-ray absorptiometry for the assessment of BMD of the lumbar spine (L1–L4), femoral neck (FN), and total body as well as body composition by the Lunar DPX-Plus device (Lunar Inc., Madison, WI, USA). Body fat percentage was categorised as decreased, normal, and increased (Table 1). The C-reactive protein (CRP) concentration was analysed in a local laboratory. We conducted analyses for three groups (UC vs. CD vs. CG) and for two groups—IBD (including UC and CD) vs. CG.

The results are presented as medians and interquartile ranges—Median [Q_1_–Q_3_] since the data did not follow a normal distribution. Normality was checked with the use of the Shapiro–Wilk test. The comparison between patients with Crohn’s disease (CD), ulcerative colitis (UC), and control group (CG) was performed with the Kruskal–Wallis test. In the case of the test of statistical significance, post-hoc Dunn’s test was used in order to find the homogenous groups. The relationship between fat mass and analysed parameters was assessed with the Spearman’s rank correlation coefficient and its significance was checked with the t-student test. Statistical analyses were conducted using Statistica13.3 (TIBCO Software Inc., Palo Alto, CA, USA, 2017; Statistica [data analysis software system], version 13, https://www.tibco.com/, accessed on 18 December 2024). All tests were considered significant at *p* < 0.05.

**Figure 1:**
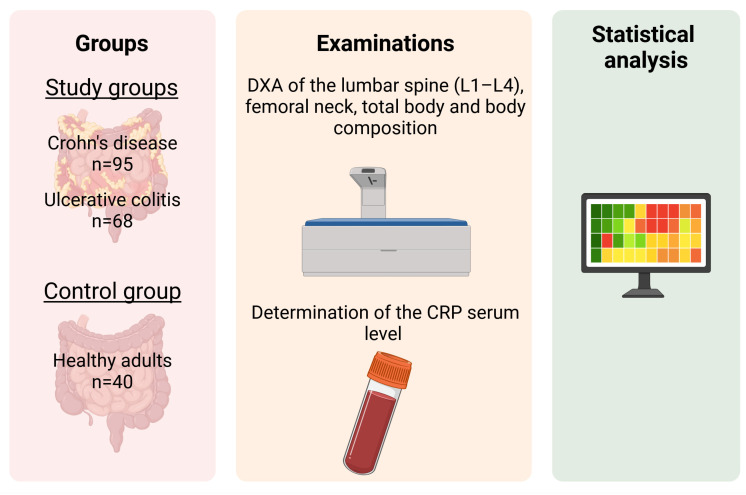
Methodology of the study (DXA—Dual-energy X-ray absorptiometry).

The summary of the methods is presented in Figure 1.

## 3. Results

We found differences in the body mass, BMI, BMD, T-score, and Z-score of femoral neck and L1–L4 as well as the CRP level between groups (Table 2). Additionally, the characteristics of the group according to the Montreal Classification are presented in Table 3 and Table 4.

A lowered fat mass percentage occurred in about 8% of CD, 13% of UC, and 3% of CG (Table 5 and Table 6). UC women had a lower body fat mass and percentage of fat mass than CD and healthy women. We did not find any differences in fat mass and percentage of fat mass among men (Table 7).

We found a positive correlation between the fat body mass and BMD and T-score of L1–L4, FN, and total body among women with CD and in a group of women with IBD (including CD and UC), but not in a group of women with CD. Fat body mass also correlated with CRP in CD and the control group in men and with BMD and T-score of L1–L4 and total body in men with UC, men with IBD (CD and UC) and the control men group, but not in the group of men with CD (Table 8).

When considering fat mass percentage, correlations were found for BMD and T-score of the femoral neck in CD and IBD women. Additionally, fat mass percentage correlated with the Z-score of total body in the group of women with IBD (including CD and UC) and with CRP in healthy women. The fat mass percentage correlated positively with the Z-score of the femoral neck in the group of men with CD and BMD and the T-score of total body in men with UC, in the group of men with IBD (including CD and UC) and control group. The fat mass percentage in healthy men correlated positively with CRP (Table 9).

The CD women with increased fat content presented a higher BMD (*p* = 0.03) and T-score (*p* = 0.03) of L1–L4 compared to women with a normal body fat percentage. Among UC men, individuals with increased fat content presented a higher BMD of the total body (*p* = 0.04) than those with normal fat percentage. However, no significant differences in BMD, T-score, or Z-score of L1–L4 and the femoral neck were found between groups stratified by decreased, normal, or increased body fat percentages (Supplemental Tables S1–S6).

## 4. Discussion

Patients with IBD exhibit lower BMI and BMD, as well as reduced T-scores and Z-scores of the femoral neck, L1–L4, and total body. These reductions are likely a consequence of the disease, as evidenced by elevated CRP levels. Inflammation affects OPG/RANKL (osteoprotegerin/system, which influences bone) [19]. Moreover, proinflammatory cytokines, among other TNF-α and IL-6 influence bone metabolism, mediating the osteocytes activity [20]. Additionally, a low BMI is a risk factor for osteoporosis [13]. Our previous studies also reported a lower BMD, T-score, and Z-score of the femoral neck and L1–L4 than healthy subjects [21,22]. Both CD and UC groups presented higher CRP levels than the healthy control group. This is consistent with the fact that inflammatory bowel diseases are chronic inflammatory conditions that contribute to elevated CRP levels.

Compared with CD or healthy women, UC women presented the lowest body fat mass and percentage of fat mass. Yadav et al. reported that disease duration in CD was not significantly associated with a decreased subcutaneous fat area and increased visceral to subcutaneous fat area ratio [5]. Bryant et al. reported reduced fat mass in 31% and 13% of CD and UC patients, respectively [23]. In a previous study, authors found no differences in the fat mass percentage between CD and UC patients [24]. We did not find differences in body fat mass among men. Women generally have higher ranges of normal fat percentages compared to men, which may explain the greater variation in fat mass loss observed in women. Additionally, estrogen concentrations in women with IBD may be decreased, influencing fat levels. Moreover, the body composition of men appears to be more stable and less susceptible to hormones and inflammatory factors.

We found some correlations between body fat mass or percentage of fat mass and bone parameters. However, these correlations remain unclear, particularly because fat tissue mass may affect bone in different ways. In women, fat tissue takes place in estrogen metabolism. Adipose tissue converts estrone sulfate to estrone and estrone to estradiol [25]. It seems important for bone health, on which there are estrogenic receptors [26].

Adipose tissue is also one of the largest endocrine organ responsible for producing many adipokines, including hormones, cytokines, extracellular matrix proteins, growth, and vasoactive factors [27]. The most common and well-known is leptin, which also may influence bone. Leptin binds to its receptor in the hypothalamus, sending a signal to osteoblasts. It decreases c-myc expression, leading to upregulating cyclin D production, which inhibits osteoblast proliferation. On the other hand, an increase in RANKL expression is observed via the PKA–ATF4 pathway, causing upregulation of osteoclast activity and bone resorption [28].

Moreover, leptin increases the expression of cocaine- and amphetamine-regulated transcript (CART), which inhibits RANKL synthesis. However, this mechanism is unknown. Additionally, leptin enhances BMSC (bone marrow mesenchymal stem cell) proliferation and differentiation to osteoblastic lineage. Leptin may affect the osteoblast directly and cause a decrease in RANKL exertion [28].

Adipose tissue produces cytokines and proinflammatory factors responsible for inflammation associated with IBD [29]. However, interestingly, Flores et al. reported that obesity is a marker of a less severe course of IBD [30]. In our study, we found a positive correlation between body fat mass and bone parameters in IBD, which may also be linked to a milder course of the disease.

A higher body fat mass and percentage of fat mass may indicate a better nutritional status. However, a systematic review by Bryant et al. found no association between body composition and disease activity [23]. Nevertheless, a higher body fat may result from more energy intake and, consequently, a higher intake of vitamins and minerals, including calcium, vitamin D and magnesium, which are essential for bone health [31].

We hypothesised that body fat mass and fat mass percentage would depend on disease exacerbation, with patients in remission presenting higher body fat mass. However, we observed a negative correlation between CRP and body fat mass only in men with CD. In the control group, CRP correlated positively with body fat mass and fat mass percentage. The previous study also demonstrated elevated CRP in adults with obesity [32]. Saijo et al. reported an association between visceral adipose tissue and CRP; however, CRP was not associated with IL-6 and TNF-α [33].

Although we carefully planned and carried out our study, there are some limitations. Firstly, we did not assess patients’ clinical status, including the duration of the disease or disease activity, nor did we determine how many patients experienced exacerbation. Our study included only CRP concentration, which represents just one parameter for assessing disease activity. Adding the Mayo scale or other measures of disease activity would improve the quality of the study. Secondly, our study focused only on body fat mass, without data on muscle mass or the distribution of fat tissue. The lack of information of medication usage is another limitation. Including these data would enhance our analysis. Future studies should assess not only BMD, but also the risk of fractures in these patients.

## 5. Conclusions

In conclusion, higher body fat mass and percentage of fat mass are likely associated with a lower risk of osteoporosis in inflammatory bowel disease. In fact, higher body fat mass or percentage of fat mass may result from a milder course of disease, positively affecting BMD. However, excessive fat mass may have an unfavourable proinflammatory effect for patients with IBD, as it could increase the risk of exacerbation. Nevertheless, maintaining good nutritional status and avoiding malnutrition are crucial for proper BMD maintenance. Future studies are needed to assess the impact of adipose tissue and the factors it releases on bone mineral density.

## Figures and Tables

**Table 1:** Range of body fat percentage.

	20–39 Years Old		40–49 Years Old	
Range	Women	Men	Women	Men
Decreased	<21%	<8%	<23%	<11%
Normal	21–32%	8–19%	23–33%	11–21%
Increased	>32%	>19%	>33%	>21%

**Table 2:** Characteristics of the groups.

Parameter	CD (n = 95)	UC (n = 68)	CG (n = 40)	*p*-Value
Age [years]	30.90 (23.90; 37.30)	32.00 (25.75; 39.40)	38.25 (27.60; 43.30)	<0.01
				<0.01 ^a^
				0.11 ^b^
				0.86 ^c^
Body mass [kg]	62.50 (55.30; 73.80)	60.85 (52.35; 76.10)	68.45 (61.60; 78.90)	0.03
				0.05 ^a^
				0.03 ^b^
				0.99 ^c^
BMI [kg/m^2^]	21.70 (19.20; 24.40)	20.33 (18.45; 24.05)	22.90 (21.25; 25.15)	<0.01
				0.12 ^a^
				<0.01 ^b^
				0.39 ^c^
BMD (L1–L4) [g/cm^2^]	1.136 (1.058; 1.217)	1.145 (1.005; 1.253)	1.246 (1.172; 1.333)	<0.0001
				<0.0001 ^a^
				<0.0001 ^b^
				0.99 ^c^
T-score (L1–L4)	−0.400 (−1.300; 0.200)	−0.400 (−1.600; 0.500)	0.400 (−0.250; 1.250)	<0.0001
				<0.0001 ^a^
				<0.0001 ^b^
				0.99 ^c^
Z-score (L1–L4)	−0.300 (−1.200; 0.400)	−0.400 (−1.300; 0.650)	0.400 (0.000; 1.050)	<0.001
				<0.001 ^a^
				<0.001 ^b^
				0.99 ^c^
BMD (femoral neck) [g/cm^2^]	1.005 (0.917; 1.145)	1.034 (0.852; 1.148)	1.094 (1.046; 1.855)	<0.01
				<0.01 ^a^
				<0.01 ^b^
				0.99 ^c^
T-score (femoral neck)	−0.300 (−1.000; 0.600)	−0.200 (−1.450; 0.700)	0.400 (0.000; 1.000)	<0.01
				<0.01 ^a^
				<0.01 ^b^
				0.99 ^c^
Z-score (femoral neck)	0.000 (−0.700; 0.800)	0.000 (−0.800; 1.050)	0.650 (0.200; 1.150)	<0.01
				<0.01 ^a^
				<0.01 ^b^
				0.99 ^c^
BMD (total body) [g/cm^2^]	1.135 (1.066; 1.193)	1.154 (1.086; 1.227)	1.227 (1.161; 1.279)	<0.0001
				<0.0001 ^a^
				<0.01 ^b^
				0.27 ^c^
T-score (total body)	−0.400 (−1.200; 0.100)	−0.100 (−1.050; 0.700)	0.500 (0.150; 1.300)	<0.0001
				<0.0001 ^a^
				<0.001 ^b^
				0.34 ^c^
Z-score (total body)	−0.100 (−1.000; 0.400)	0.100 (−0.600; 0.850)	0.600 (0.150; 1.000)	<0.0001
				<0.0001 ^a^
				0.02 ^b^
				0.08 ^c^
CRP (mg/L)	5.10 (2.00; 29.50)	4.75 (1.05; 10.55)	0.75 (0.40; 1.45)	<0.0001
				<0.0001 ^a^
				<0.0001 ^b^
				0.52 ^c^

CD—Crohn’s disease; UC—ulcerative colitis; CG—control group; BMI—body mass index; BMD—bone mineral density; CRP—C-reactive protein; ^a^—CD vs. CG; ^b^—UC vs. CG; ^c^—CD vs. UC.

**Table 3:** Number of patients with ulcerative colitis presented according to the Montreal classification [18].

Classification	Severity	Extent
0	15	-
1	18	15
2	15	19
3	20	34

**Table 4:** Number of patients with Crohn’s disease presented according to the Montreal classification [18].

Classification	Localisation	Age	Behaviour
1	15	20	34 (8)
2	30	70	18 (1)
3	50	3	13 (6)

( )—Number of patients with a perianal modifier.

**Table 5:** Number of patients with decreased, normal, and increased body fat percentages among women.

Body Fat Percentage	CD	UC	CG
Decreased	4 (8.0%)	6 (19.4%)	0
Normal	20 (40.0%)	19 (61.2%)	9 (40.9%)
Increased	26 (52.0%)	6 (19.4%)	13 (59.1%)

**Table 6:** Number of patients with decreased, normal, and increased body fat percentages among men.

Body Fat Percentage	CD	UC	CG
Decreased	4 (8.8%)	3 (8.1%)	1 (5.5%)
Normal	16 (35.6)	12 (32.4%)	6 (4.8%)
Increased	25 (55.6%)	22 (59.5%)	11 (61.1%)

**Table 7:** Body fat mass and percentage of fat mass in groups.

	WOMEN				MEN			
Parameter	CD (n = 50)	UC (n = 31)	CG (n = 22)	*p*-Value	CD (n = 45)	UC (n = 37)	CG (n = 18)	*p*-Value
Body fat mass [g]	19,274.00 (13,782.00; 23,826.00)	12,948.00 (10,604.00; 17,530.00)	22,428.00 (19,955.00; 23,849.00)	<0.0001 0.19 ^a^ <0.0001 ^b^ <0.01 ^c^	14,270.00 (7704.00; 21,382.00)	15,992.00 (9310.00; 20,710.00)	16,572.50 (12,496.00; 21,143.00)	0.74
Percentage of fat mass [%]	33.39 (33.39; 28.09)	25.85 (21.28; 31.01)	33.68 (31.06; 37.66)	<0.001 0.99 ^a^ <0.001 ^b^ <0.01 ^c^	20.73 (13.15; 26.48)	21.72 (12.77; 26.20)	21.63 (15.68; 26.83)	0.85

CD—Crohn’s disease; UC—ulcerative colitis; CG—control group; ^a^—CD vs. CG; ^b^—UC vs. CG; ^c^—CD vs. UC.

**Table 8:** Correlation between body fat mass and certain parameters among women and men.

	Women				Men			
Parameters	CD (n = 50)	UC (n = 31)	IBD (n = 81)	CG (n = 22)	CD (n = 45)	UC (n = 37)	IBD (n = 82)	CG (n = 18)
BMD (L1–L4) [g/cm^2^]	r = 0.33; *p* = 0.02	r = 0.22; *p* < 0.23	r = 0.26; *p* = 0.02	r = 0.32; *p* = 0.15	r = 0.11, *p* = 0.48	r = 0.35; *p* = 0.04	r = 0.22; *p* = 0.04	r = 0.50; *p* = 0.03
T-score (L1–L4)	r = 0.33, *p* = 0.02	r = 0.24; *p* < 0.19	r = 0.27; *p* = 0.01	r = 0.31; *p* = 0.16	r = 0.10; *p* = 0.50	r = 0.33; *p* = 0.04	r = 0.22; *p* = 0.048	r = 0.46; *p* = 0.05
Z-score (L1–L4)	r = −0.02; *p* = 0.91	r = 0.01; *p* = 0.96	r = −0.01; *p* = 0.91	r = 0.07; *p* = 0.76	r = −0.25; *p* = 0.10	r = 0.04; *p* = 0.80	r = −0.12; *p* = 0.30	r = 0.16; *p* = 0.52
BMD (femoral neck) [g/cm^2^]	r = 0.37; *p* < 0.01	r = 0.28; *p* = 0.13	r = 0.33; *p* < 0.01	r = 0.07; *p* = 0.77	r = −0.04, *p* = 0.78	r = 0.29; *p* = 0.08	r = 0.11; *p* = 0.31	r = 0.34; *p* = 0.17
T-score (femoral neck)	r = 0.36; *p* = 0.01	r = 0.28; *p* = 0.13	r = 0.33; *p* < 0.01	r = 0.08; *p* = 0.74	r = −0.07, *p* = 0.65	r = 0.29, *p* = 0.08	r = 0.10; *p* = 0.38	r = 0.35; *p* = 0.16
Z-score (femoral neck)	r = 0.24; *p* = 0.08	r = 0.10; *p* = 0.59	r = 0.19; *p* = 0.09	r = −0.16; *p* = 0.49	r = −0.26; *p* = 0.09	r = 0.19; *p* = 0.25	r = −0.03; *p* = 0.73	r = 0.17; *p* = 0.49
BMD (total body) [g/cm^2^]	r = 0.32; *p* = 0.02	r = 0.32; *p* = 0.08	r = 0.26; *p* = 0.02	r = 0.18; *p* = 0.42	r = 0.22; *p* = 0.14	r = 0.52; *p* < 0.001	r = 0.37; *p* < 0.001	r = 0.55; *p* = 0.02
T-score (total body)	r = 0.32; *p* = 0.02	r = 0.31; *p* = 0.08	r = 0.26; *p* = 0.02	*p* = 0.18; *p* = 0.43	r = 0.21; *p* = 0.16	r = 0.52; *p* < 0.01	r = 0.35; *p* < 0.01	r = 0.56; *p* = 0.02
Z-score (total body)	r = −0.19; *p* = 0.18	r = −0.03; *p* = 0.83	r = −0.21; *p* = 0.06	r = −0.25; *p* = 0.26	r = −0.21; *p* = 0.16	r = 0.25; *p* = 0.13	r = −0.01; *p* = 0.90	r = 0.12; *p* = 0.63
CRP	r = −0.01, *p* = 0.93	r = 0.16; *p* = 0.38	r = 0.09; *p* = 0.42	r = 0.76; *p* < 0.0001	r = −0.30; *p* = 0.04	r = −0.07; *p* = 0.68	r = −0.17; *p* = 0.12	r = 0.61; *p* < 0.01

CD—Crohn’s disease; UC—ulcerative colitis; CG—control group; BMD—bone mineral density; CRP—C-reactive protein.

**Table 9:** Correlation between the percentage of fat mass and certain parameters among women and men.

	Women				Men			
Parameter	CD (n = 50)	UC (n = 31)	IBD	CG (n = 22)	CD (n = 45)	UC (n = 37)	IBD (n = 82)	CG (n = 18)
BMD (L1–L4) [g/cm^2^]	r = 0.27; *p* = 0.06	r = 0.15; *p* = 0.41	r = 0.22; *p* = 0.05	r = 0.26; *p* = 0.24	r = 0.03; *p* = 0.87	r = 0.17; *p* = 0.31	r = 0.11; *p* = 0.33	r = 0.46; *p* = 0.06
T-score (L1–L4)	r = 0.27; *p* = 0.05	r = 0.17; *p* = 0.37	r = 0.22; *p* = 0.046	r = 0.26; *p* = 0.25	r = 0.02; *p* = 0.89	r = 0.16; *p* = 0.34	r = 0.10; *p* = 0.36	r = 0.43; *p* = 0.07
Z-score (L1–L4)	r = −0.03, *p* = 0.85	r = −0.02; *p* = 0.91	r = −0.3; *p* = 0.78	r = 0.06; *p* = 0.80	r = −0.28; *p* = 0.07	r = −0.9; *p* = 0.58	r = −0.18; *p* = 0.10	r = 0.20; *p* = 0.43
BMD (femoral neck) [g/cm^2^]	r = 0.33; *p* = 0.02	r = 0.14; *p* = 0.45	r = 0.27; *p* = 0.01	r = −0.15; *p* = 0.50	r = −0.12; *p* = 0.45	r = 0.14; *p* = 0.41	r = 0.004; *p* = 0.97	r = 0.28; *p* = 0.27
T-score (femoral neck)	r = 0.32; *p* = 0.02	r = 0.14; *p* = 0.45	r = 0.27; *p* = 0.01	r = −0.16; *p* = 0.49	r = −0.15; *p* = 0.32	r = 0.13; *p* = 0.43	r = −0.02; *p* = 0.88	r = 0.28; *p* = 0.26
Z-score (femoral neck)	r = 0.22; *p* = 0.12	r = −0.02; *p* = 0.91	r = 0.15; *p* = 0.18	−0.35; *p* = 0.11	r = −0.30, *p* = 0.04	r = 0.04; *p* = 0.80	r = −0.13; *p* = 0.24	r = 0.10; *p* = 0.70
BMD (total body) [g/cm^2^]	r = 0.25; *p* = 0.09	r = 0.20; *p* = 0.29	r = 0.17; *p* = 0.13	r = 0.13; *p* = 0.58	r = 0.11; *p* = 0.47	r = 0.38; *p* = 0.02	r = 0.25; *p* = 0.03	r = 0.52; *p* = 0.03
T-score (total body)	r = 0.25; *p* = 0.08	r = 0.19; *p* = 0.31	r = 0.17; *p* = 0.13	r = 0.12; *p* = 0.59	r = 0.10; *p* = 0.51	r = 0.36; *p* = 0.03	r = 0.23; *p* = 0.04	r = 0.53; *p* = 0.02
Z-score (total body)	r = −0.19; *p* = 0.18	r = −0.11; *p* = 0.54	r = −0.25; *p* = 0.03	r = −0.20; *p* = 0.36	r = −0.27; *p* = 0.07	r = 0.12; *p* = 0.47	r = −0.10; *p* = 0.38	r = 0.21; *p* = 0.40
CRP	r = −0.01; *p* = 0.92	r = 0.20; *p* = 0.28	r = 0.09; *p* = 0.41	r = 0.74; *p* < 0.0001	r = −0.22; *p* = 0.14	r = −0.05; *p* = 0.78	r = −0.13; *p* = 0.24	r = 0.67; *p* < 0.01

CD—Crohn’s disease; UC—ulcerative colitis; CG—control group; BMD—bone mineral density; CRP—C-reactive protein.

## Data Availability

Data and materials are available after contact with the corresponding author.

## Supplementary Materials

The following supporting information can be downloaded at: https://www.mdpi.com/article/10.3390/nu17030466/s1, Table S1: Comparing BMD, T-score and Z-score of femoral neck and lumbar spine (L1–L4) among women with Crohn’s disease with decreased, normal and increased body fat percentage; Table S2: Comparing BMD, T-score and Z-score of femoral neck and lumbar spine (L1–L4) among women with ulcerative colitis with decreased, normal and increased body fat percentage. Table S3: Comparing BMD, T-score and Z-score of femoral neck and lumbar spine (L1–L4) among healthy women with decreased, normal and increased body fat percentage. Table S4: Comparing BMD, T-score and Z-score of femoral neck and lumbar spine (L1–L4) among men with Crohn’s disease with decreased, normal and increased body fat percentage. Table S5: Comparing BMD, T-score and Z-score of femoral neck and lumbar spine (L1–L4) among men with ulcerative colitis with decreased, normal and increased body fat percentage. Table S6: Comparing BMD, T-score and Z-score of femoral neck and lumbar spine (L1–L4) among healthy men with decreased, normal and increased body fat percentage.

## Abbreviations

The following abbreviations are used in this manuscript:

IBD	Inflammatory bowel disease
CD	Crohn disease
UC	Ulcerative colitis
BMD	Bone mineral density
BMI	Body mass index
CRP	C-reactive protein
