# Supplementary material for: Is There Any Association Between Fat Body Mass and Bone Mineral Density in Patients with Crohn’s Disease and Ulcerative Colitis?
Source: Nutrients. 2025 Jan 28;17(3):466. doi: 10.3390/nu17030466 (PMC11820439; doi:10.3390/nu17030466)
Supplement: Supplementary file 1 [file nutrients-17-00466-s001.zip › nutrients-3410267-supplementary.pdf]

**Suplemental Table S1.** Comparing BMD, T-score and Z-score of femoral neck and lumbar spine (L1-L4) among women with Crohn's disease with decreased, normal and increased body fat percentage

| | Decreased (n=4) | Normal (n=20) | Increased (n=26) | p-value |
|---|---|---|---|---|
| BMD (L1-L4) [g/cm$^2$] | 1.19 (1.10; 1.23) | 1.11 (0.98;1.18) | 1.19 (1.10; 1.28) | 0.03<br>0.69[a]<br>0.99[b]<br>0.03[c] |
| T-score (L1-L4) | 0.05 (-0.70; 0.40) | -0.55 (-1.65; 0.05) | 0.10 (-0,60; 0.80) | 0.03<br>0.71[a]<br>0.99[b]<br>0.03[c] |
| Z-score (L1-L4) | 0.70 (0.05; 0.85) | -0.25 (-1.05; 0.20) | -0.10 (-0.50; 0.80) | 0.2 |
| BMD (femoral neck) [g/cm$^2$] | 0.94 (0.83; 1.08) | 0.94 (0.82;1.04) | 1.08 (0.95; 1.16) | 0.08 |
| T-score (femoral neck) | -0.70 (-1.50; 0.30) | -0,70 (-1.60; 0.05) | 0.30 (-0.60; 0.90) | 0.09 |
| Z-score (femoral neck) | 0.05 (-0.50; 1.05) | -0.10 (-1.30; 0.55) | 0.40 (-0.30; 1.50) | 0.19 |

a- Decreased vs normal; b- decreased vs increased; c- increased vs normal

**Suplemental Table S2.** Comparing BMD, T-score and Z-score of femoral neck and lumbar spine (L1-L4) among women with ulcerative colitis with decreased, normal and increased body fat percentage

| | Decreased (n=6) | Normal (n=19) | Increased (n=6) | p-value |
|---|---|---|---|---|
| BMD (L1-L4) [g/cm$^2$] | 1.09 (0.94; 1.31) | 1.11 (0.99; 1,26) | 1.26 (1.15; 1.32) | 0.32 |
| T-score (L1-L4) | -0.70 (-2.00; 1.10) | -0.60 (-1.60; 0,70) | 0.70 (-0.20; 1.20) | 0.26 |
| Z-score (L1-L4) | -0.10 (-1.10; 1.40) | -0.40 (-1.30; 1.00) | 0.55 (-0.20; 1.40) | 0.82 |
| BMD (femoral neck) [g/cm$^2$] | 0.95 (0.80; 1.06) | 0.91 (0.80; 1.11) | 1.04 (0.97; 1.09) | 0.81 |
| T-score (femoral neck) | -0.65 (-1.70; 0.20) | -1.00 (-1.70; 0.50) | 0.05 (-0.50; 0.40) | 0.82 |
| Z-score (femoral neck) | -0.05 (-0.90; 0.80) | -0.40 (-1.00; 1.00) | 0.20 (-0.80; 0.60) | 0.97 |

**Suplemental Table S3.** Comparing BMD, T-score and Z-score of femoral neck and lumbar spine (L1-L4) among healthy women with decreased, normal and increased body fat percentage

| | Normal (n=9) | Increased (n=13) | p-value |
|---|---|---|---|
| BMD (L1-L4) [g/cm$^2$] | 1.22 (1.17; 1.34) | 1.26 (1.20; 1.30) | 0.99 |
| T-score (L1-L4) | 0.40 (-0.10; 1.30) | 0.70 (0.20; 1.00) | 0.99 |
| Z-score (L1-L4) | 0.40 (0.30; 1.60) | 0.40 (0.20; 1.20) | 0.99 |
| BMD (femoral neck) [g/cm$^2$] | 1.09 (1.04; 1.14) | 1.09 (1.05; 1.16) | 0.99 |
| T-score (femoral neck) | 0.40 (0.00; 0.70) | 0.40 (0.10; 0.90) | 0.99 |
| Z-score (femoral neck) | 0.80 (0.40; 1.20) | 0.40 (0.10; 1.10) | 0.99 |

**Suplemental Table S4.** Comparing BMD, T-score and Z-score of femoral neck and lumbar spine (L1-L4) among men with Crohn's disease with decreased, normal and increased body fat percentage

| | Decreased (n=4) | Normal (n=16) | Increased (n=25) | p-value |
|---|---|---|---|---|
| **BMD (L1-L4) [g/cm$^2$]** | 1.11 (0.88; 1.19) | 1.13 (1.08; 1.20) | 1.10 (1.06; 1.20) | 0.73 |

| | | | | |
|---|---|---|---|---|
| **T-score (L1-L4)** | -0.95 (-2.90; -0.30) | -0.65 (-1.20; -0.10) | -1.00 (-1.30; -0.20) | 0.70 |
| **Z-score (L1-L4)** | 0.25 (-1.55; 0.65) | -0.30 (-0.70; 0.15) | -1.20 (-1.60; -0.10) | 0.21 |
| **BMD (femoral neck) [g/cm²]** | 1.16 (0.96; 1.32) | 1.08 (0.97; 1.13) | 1.02 (0.99; 1.08) | 0.55 |
| **T-score (femoral neck)** | 0.65 (-0.90; 1.95) | 0.00 (-0.80; 0.50) | -0.40 (-0.60;-0.10) | 0.41 |
| **Z-score (femoral neck)** | 1.00 (-0.35; 2.40) | 0.05 (-0.45; 0.70) | -0.50 (-0.70; 0.20) | 0.17 |

**Suplemental Table S5.** Comparing BMD, T-score and Z-score of femoral neck and lumbar spine (L1-L4) among men with ulcerative colitis with decreased, normal and increased body fat percentage

| | **Decreased (n=3)** | **Normal (n=12)** | **Increased (n=22)** | **p-value** |
|---|---|---|---|---|
| BMD (L1-L4) [g/cm²] | 1.18 (0.99; 1.38) | 1.08\9 (0.90; 1.20) | 1.20 (1.06; 1.25) | 0.24 |
| T-score (L1-L4) | -0.30 (-1.90; 1.40) | -1.00 (-2.65; -0.15) | -0.15 (-1.30; 0.20) | 0.28 |
| Z-score (L1-L4) | 0.50 (-1.00; 1.80) | -0.90 (-2.05; 0.05) | -0.55 (-1.30; -0.10) | 0.32 |
| BMD (femoral neck) [g/cm²] | 1.03 (0.92; 1.21) | 1.02 (0.82; 1.20) | 1.10 (0.96; 1.26) | 0.59 |
| T-score (femoral neck) | -0.30 (-1.20; 1.00) | -0.25 (-1.95; 1.05) | 0.20 (-0.90; 1.60) | 0.63 |
| Z-score (femoral neck) | 0.00 (-0.70; 1.20) | -0.25 (-1.40; 1.35) | 0.10 (-0.70; 1.50) | 0.85 |

**Suplemental Table S6.** Comparing BMD, T-score and Z-score of femoral neck and lumbar spine (L1-L4) among healthy men with decreased, normal and increased body fat percentage

| | **Decreased (n=1)** | **Normal (n=6)** | **Increased (n=11)** | **p-value** |
|---|---|---|---|---|
| BMD (L1-L4) [g/cm²] | 1.18 | 1.16 (1.13; 1.27) | 1.28 (1.17; 1.34) | 0.26 |
| T-score (L1-L4) | -0.40 | -0.35 (-0.50; 0.40) | 0.50 (-0.40; 1.20) | 0.31 |
| Z-score (L1-L4) | 0.20 | -0.10 (-0.20; 0.50) | 0.40 (0.00; 1.00) | 0.66 |
| BMD (femoral neck) [g/cm²] | 1.18 | 1.08 (1.02; 1.17) | 1.16 (1.09; 1.23) | 0.30 |
| T-score (femoral neck) | 0.80 | 0.05 (-0.40; 0.70) | 0.70 (0.10; 1.30) | 0.26 |
| Z-score (femoral neck) | 1.10 | 0.45 (0.00; 0.60) | 0.80 (0.20; 1.40) | 0.50 |
